# Morphometric Analysis of Accessory Palatine Canals in Human Dry Skulls: Clinical Implications for Maxillofacial Surgery

**DOI:** 10.7759/cureus.77357

**Published:** 2025-01-13

**Authors:** P. Yeshwanthi, Nikhil Aggarwal, Ajay Kumar Verma, Archana Rani, Jyoti Chopra, Punita Manik

**Affiliations:** 1 Anatomy, King George's Medical University, Lucknow, IND; 2 Anatomy, Maulana Azad Medical College, New Delhi, IND

**Keywords:** anaesthesia, anatomy, cbct, implantology, maxilla, morphometry, neurovascular, palate, skulls, surgery

## Abstract

Introduction

The premaxilla, located anterior to the incisive canal and including the central and lateral incisors, plays a crucial role in oral and maxillofacial surgeries due to its neurovascular structures. Accessory palatine canals are of clinical importance as they help prevent complications such as anesthesia failure and nerve injury. While many radiological studies have examined these canals, research on dried skulls remains scarce. This study aims to offer detailed anatomical insights into accessory palatine canals, with the goal of enhancing surgical precision and minimizing complications in palatal surgeries.

Aim

The aim of this study is to determine the prevalence of accessory palatine canals and conduct a morphometric analysis of these canals in relation to the incisive canal in the premaxilla of human dry skulls.

Materials and methods

This cross-sectional observational study was conducted in the Department of Anatomy at King George’s Medical University, Lucknow, India, from April 2022 to September 2023. A total of 100 dry human skulls, with unknown age and sex, were examined for the presence of accessory palatine canals, excluding the nasopalatine, greater palatine, and lesser palatine canals. Skulls with damaged, broken, or eroded maxillae were excluded from the study. The canals were analyzed for their length, vertical and horizontal diameters, and distance from the incisive foramen. All measurements were taken using the divider-scale method and a digital vernier caliper. The data were organized in an Excel file and analyzed using IBM SPSS Statistics for Windows, Version 26.0 (Released 2019; IBM Corp., Armonk, NY, USA). The frequency of occurrence of these accessory canals, along with the mean and SD, were calculated.

Results

Accessory palatine canals were observed in 10 out of 100 skulls. Among these, six skulls had one accessory canal, two had two canals each, and the remaining two skulls had three and four accessory palatine canals, respectively. The majority of these canals opened in the central incisor socket (70.6%), while the rest opened in the lateral incisor socket (29.4%). The mean length of the canal (3.7 ± 3 mm) could be measured in 12 of the 17 canals, as five canals were visible only as openings. The mean vertical diameter was 1.4 ± 0.9 mm, the horizontal diameter was 2 ± 1 mm, and the mean distance from the incisive canal was 6.5 ± 1.3 mm.

Conclusions

Although the incidence of accessory palatine canals is low, these canals can present challenges during palatine reconstructive surgeries by altering the neurovascular anatomy, thus increasing the risk of complications such as bleeding or nerve injury. A thorough understanding of the morphometry of these canals is essential for surgeons, as it enables better management of palatal surgeries for both pathologies and the reconstruction of the region.

## Introduction

The field of oral and maxillofacial surgery has seen significant advancements, leading to an increase in the number of surgeries performed on the maxilla. This rise in surgical procedures necessitates a deeper understanding of the normal and variant anatomy of the anterior maxilla to prevent neurovascular injuries and improve surgical outcomes [1,2]. While the incisive canal and foramen, which transmit the nasopalatine nerve and artery, have been extensively studied, there is limited research on the accessory palatine canal [3]. The maxilla, a key structure in the craniofacial complex, contains several critical neurovascular components. The incisive canal, or nasopalatine canal (NPC), is a well-documented structure that facilitates the passage of the nasopalatine nerve and artery from the nasal cavity to the oral cavity [4]. Previous studies have shown that the prevalence of accessory canals varies widely among individuals, with some populations exhibiting a higher frequency of these structures [5,6].

Understanding anatomical variations in accessory palatine canals is essential for clinicians involved in dental implantology, maxillofacial surgery, and endodontics. Unintentional damage to the neurovascular bundles within these canals during surgical procedures can lead to serious complications, including excessive bleeding, pain, and delayed healing. This risk is particularly high for accessory canals, which often contain branches of the greater palatine nerve and artery. Injury to these structures can result in significant postoperative issues, such as hemorrhage and prolonged healing, underscoring the importance of preoperative imaging and careful surgical planning [7]. For example, studies have demonstrated that cone beam CT (CBCT) imaging can detect accessory canals and their neurovascular contents, allowing clinicians to avoid potentially hazardous regions during procedures like sinus lifts or implant placements [8]. Detailed morphometric data on these canals enable surgeons to plan and perform safer surgeries by providing clear anatomical landmarks to minimize risks [9].

The anatomical complexity of the premaxilla is further compounded by variations in the course and branching patterns of nerves and blood vessels. The anterior superior alveolar nerve and artery, branching from the infraorbital nerve and artery, respectively, traverse the bone to innervate and supply the maxillary teeth. Variations in their pathways can lead to additional foramina and canals that may be overlooked during clinical evaluations [10]. Furthermore, the presence of accessory canals significantly affects the efficacy of local anesthesia in dental procedures. An incomplete understanding of these variations may result in ineffective anesthesia, leading to patient discomfort and procedural challenges [11]. To address this gap, this study provides a detailed morphometric analysis of accessory palatine canals using human dry skulls, documenting their prevalence, dimensions, and spatial relationships to the incisive canal. The findings aim to assist clinicians in enhancing the precision and safety of surgical interventions in the premaxillary region.

## Materials and methods

Study design and setting

This cross-sectional observational study was conducted in the Department of Anatomy at King George’s Medical University, Lucknow, India, from April 2022 to September 2023. The aim of the study was to assess and analyze the morphometry of accessory palatine canals in dry human skulls. The study adhered to the ethical guidelines set by the institution, and an informed consent waiver was granted by the Institutional Ethical Committee (ref. code: XIX-PGTSC-IIA/P50).

Sample selection and inclusion criteria

A total of 100 dry human skulls were included in the study, selected from the osteology section of the university, with unknown age, sex, and ethnicity. Only well-preserved skulls with intact maxillary structures were included. Skulls exhibiting visible fractures, significant erosion, or any damage that could interfere with the accurate assessment of canal morphology were excluded. This selection process aimed to ensure consistency in measurements and minimize variability caused by damaged specimens.

Measurement techniques and tools

**Figure 1:**
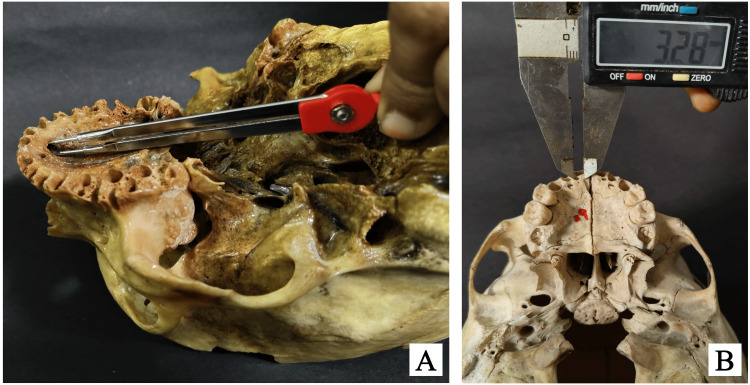
Images of skulls illustrating the measurement techniques: (A) using the divider-scale method and (B) using a standard digital caliper

**Figure 2:**
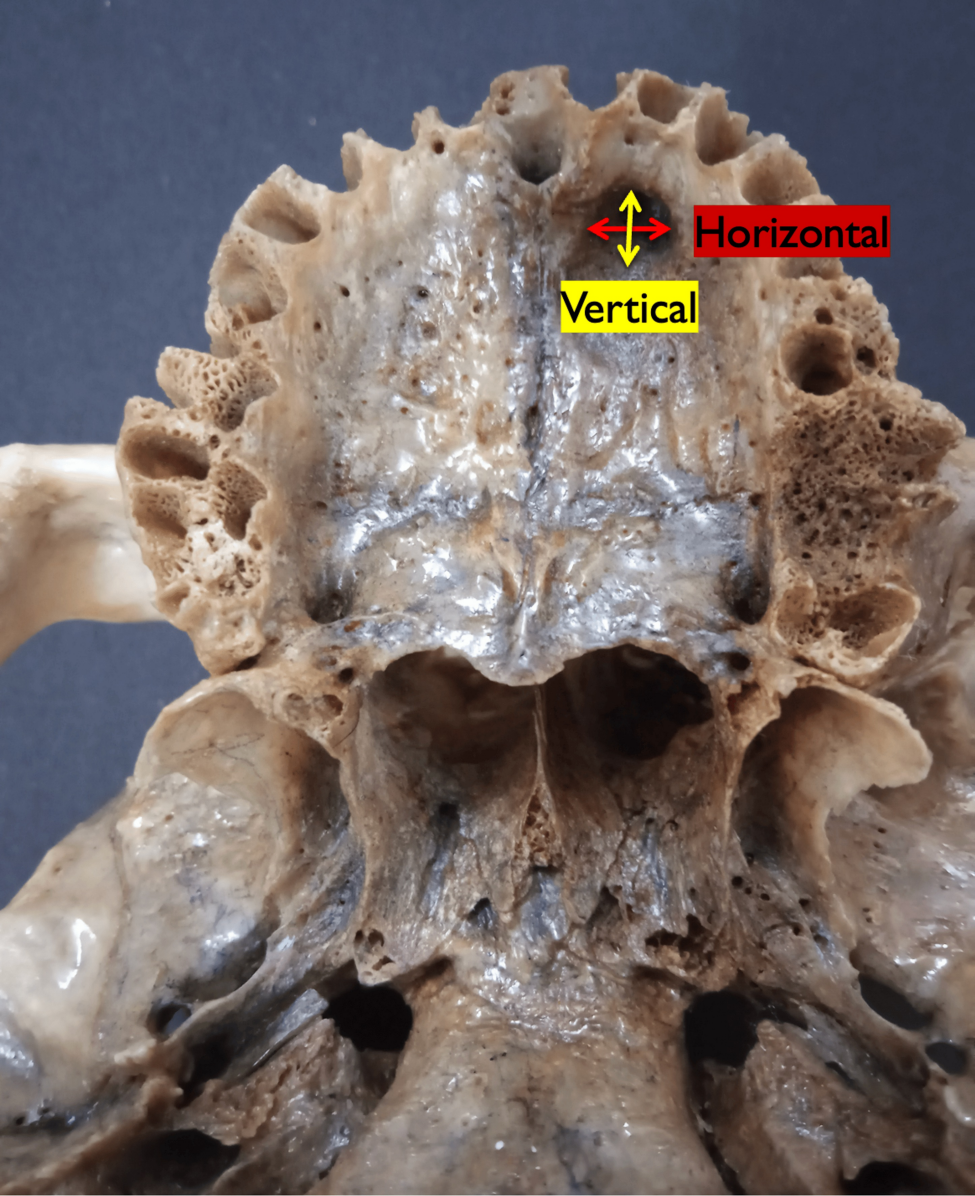
Image of a skull showing the measurements of the vertical diameter (yellow arrow) and horizontal diameter (red arrow) of the palatal opening of the accessory palatine canals

**Figure 3:**
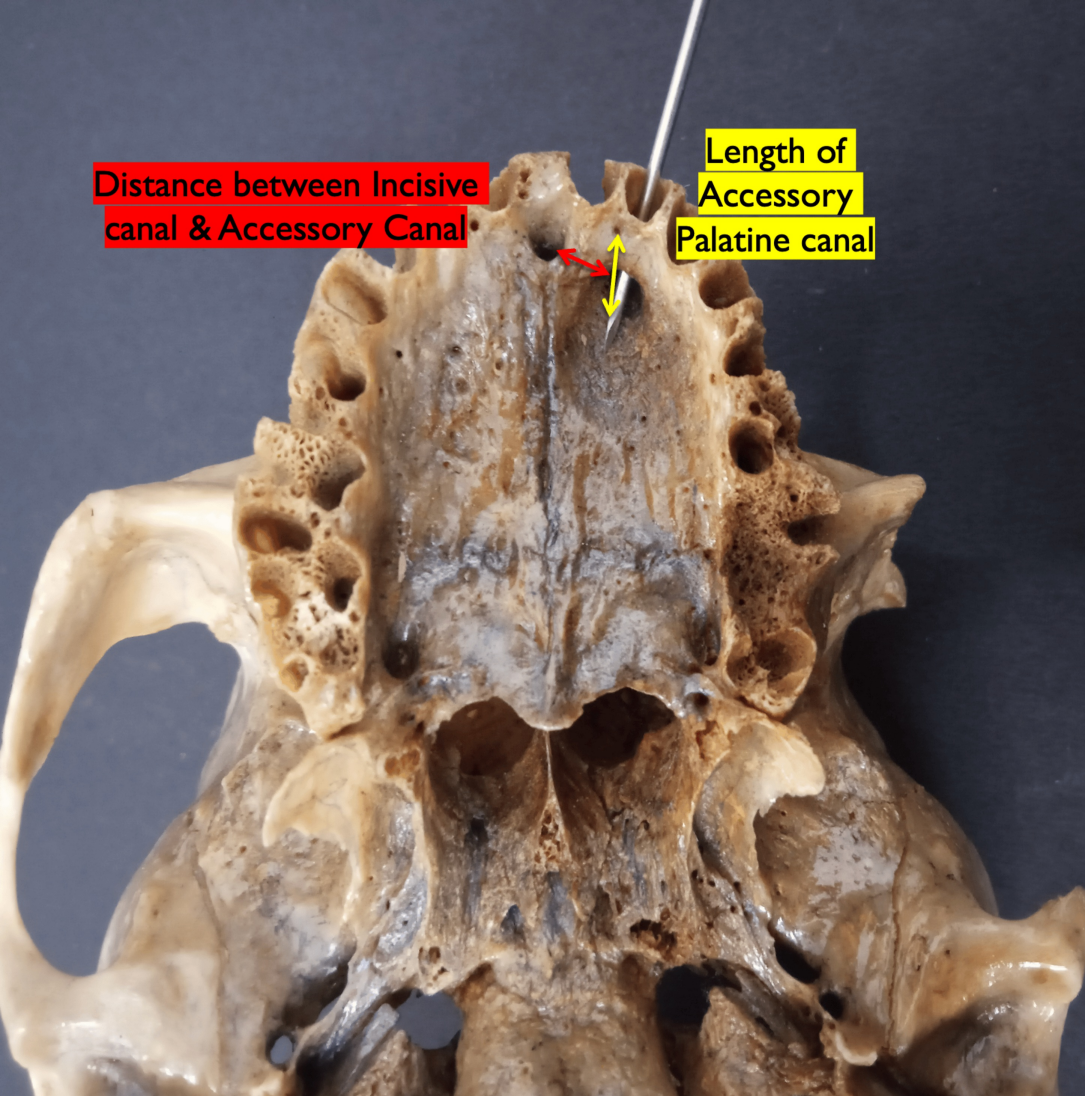
Image of a skull showing the measurements of the length of the palatal opening of the accessory canals (yellow arrow) and the distance of the accessory canal from the incisive canal in the midline (red arrow)

The morphometric analysis was performed using both the divider-scale method (Figure 1A) and a digital vernier caliper (Figure 1B). The divider-scale method was initially used for approximate measurements of lengths and diameters, while the digital caliper, with a precision of 0.01 mm, was employed for more accurate final readings. The vertical diameter of the palatal opening of each accessory palatine canal was measured from the superior to the inferior margin of the canal opening, providing an assessment of its height (Figure 2). The horizontal diameter was measured across the widest dimension from side to side to capture the canal’s width (Figure 2). The length of the palatal opening was recorded from the palatal opening to its endpoint within the maxillary bone, representing the canal’s total internal distance (Figure 3). Additionally, the distance from the accessory canal to the incisive canal in the midline was measured to establish its spatial relationship within the premaxilla (Figure 3). To minimize measurement errors and ensure consistency, each parameter was measured three times for each canal, and the average of these three measurements was used for analysis. The measurements were taken by two independent observers, and inter-observer variability was assessed to ensure reliability.

Data collection procedures

The examination was conducted systematically, with each skull positioned on a standardized orientation platform to ensure uniformity during measurements. Any canals located within the anterior maxilla, excluding the well-documented nasopalatine, greater palatine, and lesser palatine canals, were classified as accessory palatine canals. The palatal openings of the canals were photographed and labeled for documentation, and measurements of their visible and accessible portions were recorded on a standardized data sheet.

Data analysis

Data collected during the study period were entered into IBM SPSS Statistics for Windows, Version 26.0 (Released 2019; IBM Corp., Armonk, NY, USA) for statistical analysis. Descriptive statistics, including mean, SD, and frequency distributions, were calculated for the morphometric parameters. The presence and characteristics of accessory palatine canals were analyzed for patterns in their distribution, dimensions, and proximity to anatomical landmarks such as the incisive canal. A bar chart and pie chart were used to visually represent the frequency and location of the canals.

## Results

**Figure 4:**
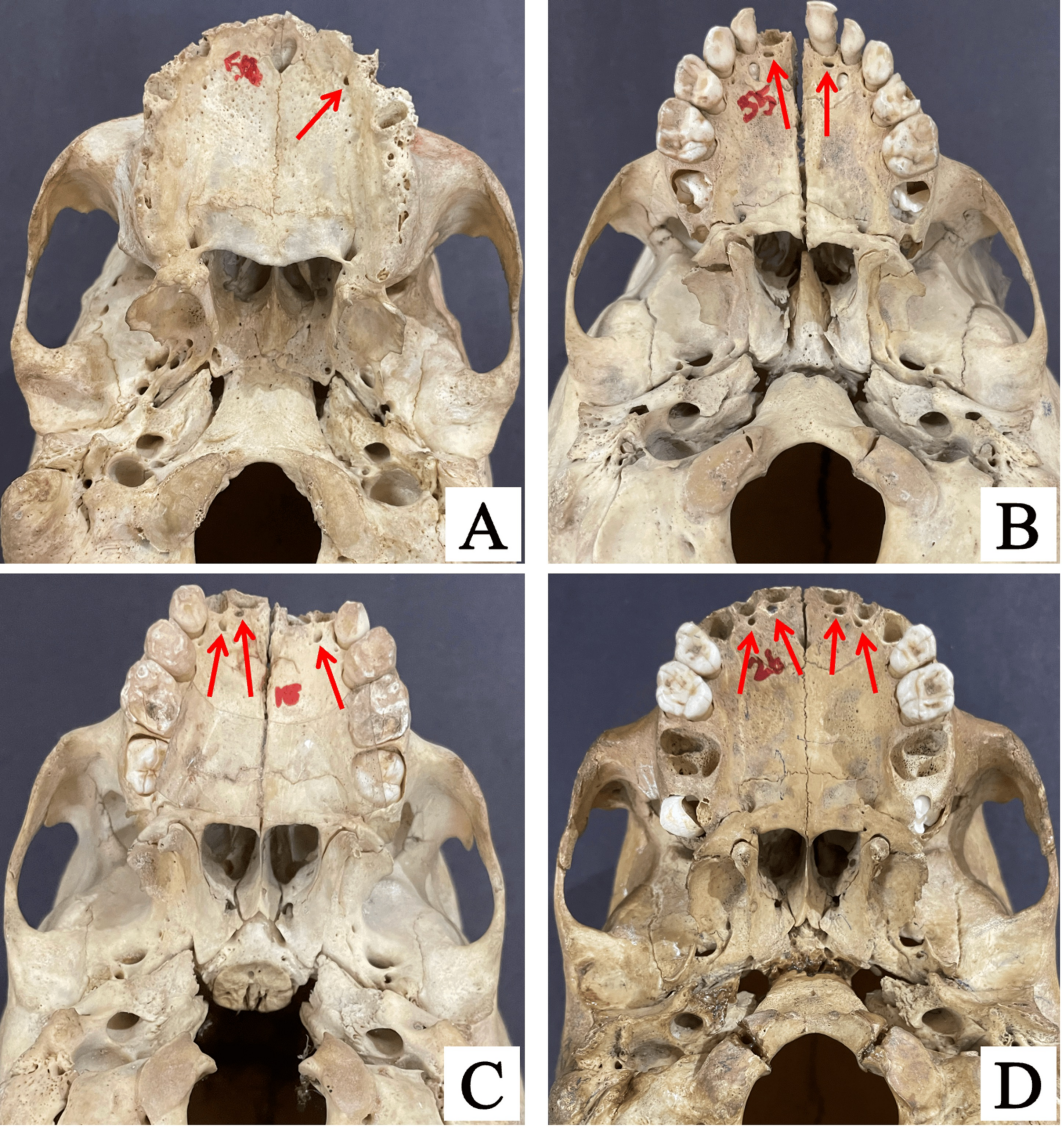
Skull images showing variations in the number of accessory palatine canals: a skull with one canal (A), two canals (B), three canals (C), and four canals (D), as indicated by red arrows in all images

Accessory palatine canals were identified in 10 out of the 100 skulls examined. The majority of the skulls with accessory palatine canals had only one canal, representing 60% of the cases. Skulls with two canals made up 20%, while those with three and four canals each accounted for 10% of the cases (Figure 4).

**Figure 5:**
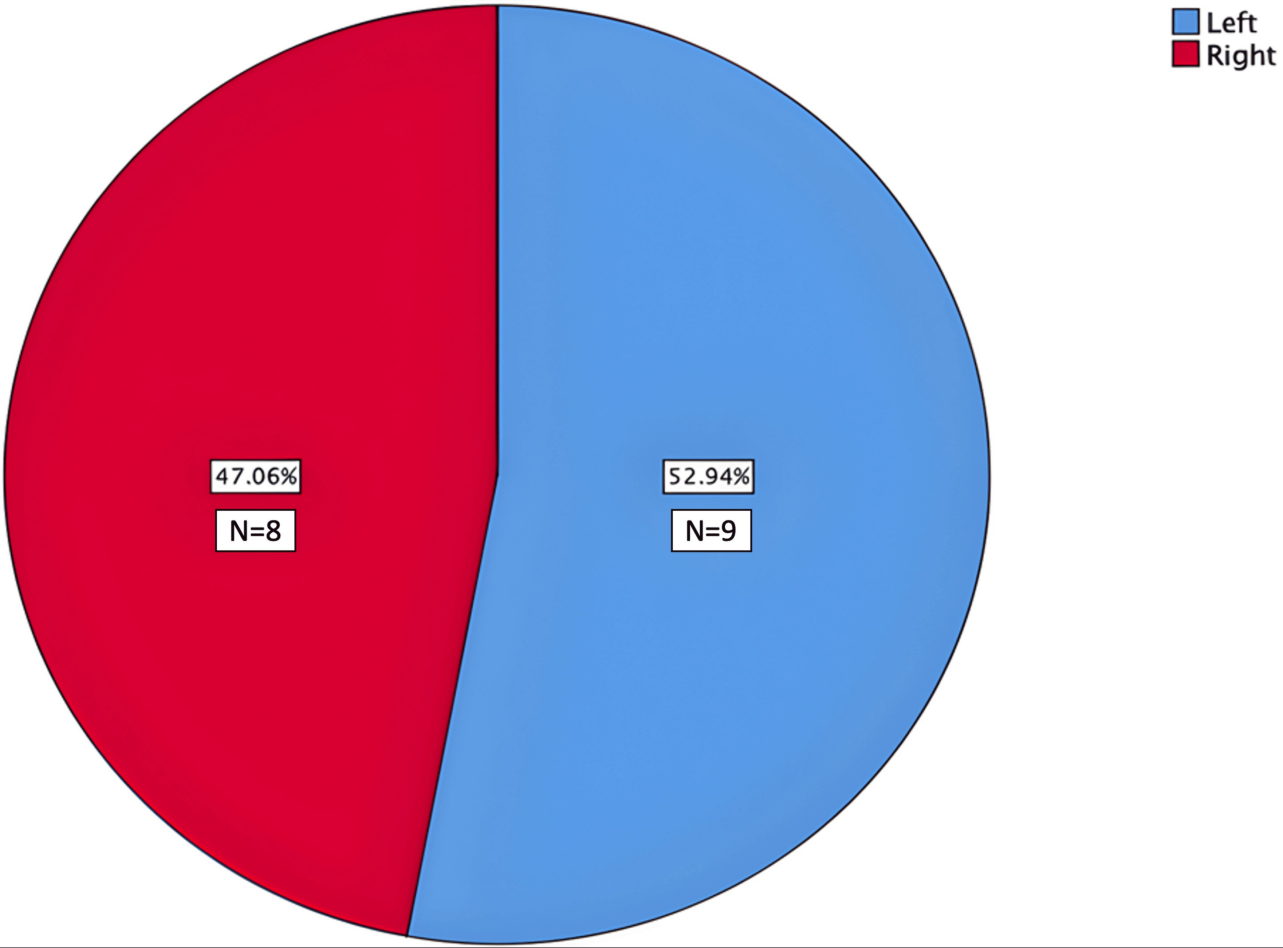
Side distribution of accessory palatine canals

A total of 17 accessory palatine canals were identified (Table 1). The canals were nearly evenly distributed between the left and right sides, with a slight predominance on the left side (52.9%). Specifically, nine canals were observed on the left side of the incisive canal (midline), while eight were noted on the right side (Figure 5).

**Table 1:** Distribution of accessory palatine canals among the 10 skulls with identified canals

Number of canals per skull	Frequency	Percentage (%)
1	6	60
2	2	20
3	1	10
4	1	10

**Figure 6:**
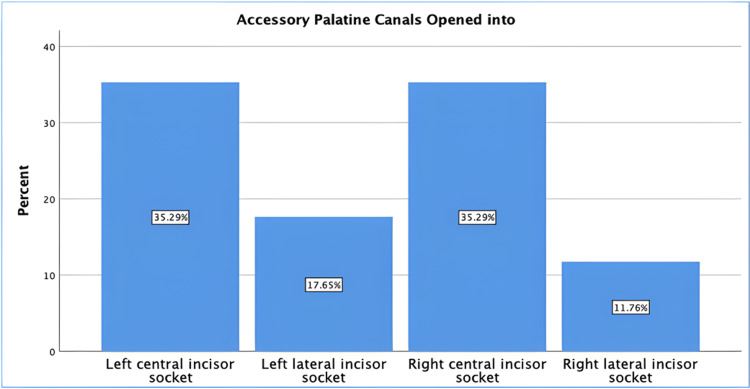
Opening location of accessory palatine canals

The majority of the accessory palatine canals (70.6%, n = 12) opened in the central incisor socket, while the remaining 29.4% (n = 5) opened in the lateral incisor socket (Figure 6).

The length of the palatal opening of a canal could be assessed in only 12 out of the 17 canals, as five canals were observed only as openings. The mean length of the palatal opening of the accessory palatine canals was 3.7 mm, with a mean vertical diameter of 1.4 mm and a horizontal diameter of 2.0 mm. The average distance from the incisive canal to the accessory canal was 6.5 mm (Table 2).

**Table 2:** Morphometric data of accessory palatine canals

Measurements	Mean ± SD
Mean length of the palatal opening (mm)	3.7 ± 3.0
Mean vertical diameter of the palatal opening (mm)	1.4 ± 0.9
Mean horizontal diameter of the palatal opening (mm)	2.0 ± 1.0
Mean distance from the incisive canal (mm)	6.5 ± 1.3

## Discussion

The anatomical variations of accessory palatine canals in the anterior maxilla have gained significant clinical attention due to their potential implications in surgery. This study identified accessory canals in 10% of the examined dry skulls, which is consistent with the reported prevalence range of 16-100% in the literature [2,12,13]. Previous radiological studies and observations in dry skulls have emphasized the importance of understanding these variations to avoid complications during maxillofacial surgeries [12]. For example, de Oliveira-Santos et al. (2013) observed additional foramina in the anterior palate in 15% of cases, with a mean diameter of 1.4 mm. This underscores the need for careful preoperative planning, especially in regions with a higher prevalence of accessory canals [1]. Similarly, von Arx et al. (2013) reported a prevalence of 27.8% for accessory canals in the anterior maxilla, with a mean diameter of 1.31 mm [13]. These findings illustrate the variability in both the occurrence and dimensions of accessory palatine canals across different populations.

The morphometric data from this study, including the average length, vertical diameter, and horizontal diameter of the accessory palatine canals, provide valuable insights for surgeons. For example, the mean length of 3.7 mm and the average distance of 6.5 mm from the incisive canal can serve as critical reference points during surgical planning. Advanced imaging techniques, such as CBCT, have proven effective in detecting accessory canals that may not be visible on standard radiographs. CBCT can enhance preoperative assessments and intraoperative navigation, reducing the risk of complications [14,15]. This technology is especially useful for identifying small or hidden canals that could otherwise be missed, potentially leading to unforeseen surgical complications.

The findings of this study align with those of other researchers examining the prevalence and characteristics of accessory palatine canals. Sekerci et al. (2015) conducted a CBCT study on a pediatric population and found that 22% of cases had additional foramina in the anterior palate, excluding the NPC, with an average diameter of 1.2 mm [16]. This study also highlights the importance of considering age-related anatomical variations, as the prevalence and dimensions of accessory palatine canals can differ across age groups.

Kikuta et al. (2019) reported a rare case of a supernumerary incisive canal in a cadaveric study, where small branches of the greater palatine artery and nerve entered the additional canal [17]. This finding emphasizes the need for comprehensive anatomical studies to identify rare but clinically significant variations. The presence of such variations underscores the importance of thorough anatomical assessments before performing surgical procedures in the anterior maxilla. Table 3 compares the findings of the present study with previously published literature.

**Table 3:** Comparison of the findings from the present study with previously published literature AC, accessory canal; APF, accessory palatine foramen; NPC, nasopalatine canal

Author	Year	Sample size	Study place	Study type	Findings
de Oliveira-Santos et al. [1]	2013	178	Belgium	CT study	Fifteen percent of the population had additional foramina in the anterior palate, with an average diameter of 1.4 mm, located in variable positions.
Vasiljevic et al. [2]	2021	130	Serbia	CT study	ACs are highly prevalent, typically presenting bilaterally and often in a curved shape, with the palatal foramen positioned accordingly.
Marzook et al. [12]	2021	170	Egypt	CT study	The anterior APF was observed in 73.53% of scans, while bilateral APF was present in 43.53% of cases.
von Arx et al. [13]	2013	176	Switzerland	CT study	In 27.8% of cases, one or more ACs, other than the NPC, were found in the anterior maxilla, with a mean diameter of 1.31 mm.
Sekerci et al. [16]	2014	368	Turkey	CT study	Twenty-two percent of the population had additional foramina, other than the NPC, in the anterior palate, with an average diameter of 1.2 mm and located in variable positions.
Kikuta et al. [17]	2019	1	Japan	Cadaver study	A supernumerary incisive canal has been reported, through which small branches of the greater palatine artery and nerve enter. This study is one of the few cadaveric/dry skull studies focusing on ACs and foramina in the anterior maxilla.
Present study	2023	100	India	Dry skull	In 10% of the skulls, additional accessory palatine canals were observed. The mean length of these canals was 3.7 ± 3 mm. The mean vertical diameter was 1.4 ± 0.9 mm, and the horizontal diameter was 2 ± 1 mm. Additionally, the distance from the AC to the incisive canal was measured at 6.5 ± 1.3 mm, providing a valuable reference for clinicians to be more cautious when working in this region.

The clinical implications of accessory palatine canals extend beyond maxillofacial surgery and dental implantology, as their presence can impact procedures such as endodontic treatments by providing potential pathways for the spread of infection. Understanding these anatomical variations in the anterior maxilla can help clinicians plan safer and more effective treatments [14,15]. Recognizing such variations is also crucial for radiologists and surgeons to minimize iatrogenic errors during clinical procedures [18].

The detailed morphometric data provided by this study offer valuable reference points for surgical planning, helping to identify accessory canals and reduce the risks of nerve damage, bleeding, or anesthesia failure. To further enhance surgical precision, we recommend the use of preoperative imaging techniques such as CBCT to map canal configurations and minimize complications during procedures like palatal reconstruction and implant placements [12].

Future research utilizing advanced imaging techniques and cadaveric dissections is needed to confirm and expand upon these findings, offering a more comprehensive understanding of the clinical implications of accessory palatine canals.

Limitations

Despite the valuable insights provided by this study, it is important to acknowledge its limitations. The use of dry skulls restricts the ability to trace the full course of neurovascular structures within the canals, preventing the detection of potential tortuous or curved configurations as described in previous studies. Additionally, the methodology is limited to measuring the visible and accessible portions of the canals, which may not account for their continuation deeper within the maxilla. Another limitation is the inability to trace and measure the length of accessory canals that follow a tortuous path within the maxilla. Moreover, the study could not assess canals or foramina smaller than 1 mm, as these would require advanced imaging techniques such as CBCT for detailed visualization. These methodological constraints may affect the direct applicability of the findings to live patients, where soft tissue dynamics and neurovascular interactions are crucial considerations. Lastly, the relatively small sample size of 100 skulls limits the generalizability of the findings, emphasizing the need for larger studies to validate these results. Future research incorporating advanced imaging techniques and live subjects could help bridge these gaps, offering a more comprehensive understanding of the clinical relevance of accessory palatine canals.

## Conclusions

This observational study provides valuable insights into the anatomical variations and morphometric characteristics of accessory palatine canals in the anterior maxilla. The findings suggest that these canals are present in a significant proportion of individuals, with variations in their dimensions and locations. By advancing our understanding of maxillary anatomy, this study contributes to improving surgical safety and patient outcomes. It also underscores the need for future research involving advanced imaging techniques, live subjects, and larger, demographically diverse samples to further validate and expand upon these findings.
